# Correlation in Causality: A Progressive Study of Hierarchical Relations within Human and Organizational Factors in Coal Mine Accidents

**DOI:** 10.3390/ijerph18095020

**Published:** 2021-05-10

**Authors:** Ziwei Fa, Xinchun Li, Quanlong Liu, Zunxiang Qiu, Zhengyuan Zhai

**Affiliations:** School of Management, China University of Mining & Technology, Xuzhou 221116, China; TB20070001B3LD@cumt.edu.cn (Z.F.); qll2016@cumt.edu.cn (Q.L.); TS20070117A31@cumt.edu.cn (Z.Q.); cumtzzy@cumt.edu.cn (Z.Z.)

**Keywords:** coal mine accidents, HFACS framework, data-driven, text mining, association rules

## Abstract

It has been revealed in numerous investigation reports that human and organizational factors (HOFs) are the fundamental causes of coal mine accidents. However, with various kinds of accident-causing factors in coal mines, the lack of systematic analysis of causality within specific HOFs could lead to defective accident precautions. Therefore, this study centered on the data-driven concept and selected 883 coal mine accident reports from 2011 to 2020 as the original data to discover the influencing paths of specific HOFs. First, 55 manifestations with the characteristics of the coal mine accidents were extracted by text segmentation. Second, according to their own attributes, all manifestations were mapped into the Human Factors Analysis and Classification System (HFACS), forming a modified HFACS-CM framework in China’s coal-mining industry with 5 categories, 19 subcategories and 42 unsafe factors. Finally, the Apriori association algorithm was applied to discover the causal association rules among external influences, organizational influences, unsafe supervision, preconditions for unsafe acts and direct unsafe acts layer by layer, exposing four clear accident-causing “trajectories” in HAFCS-CM. This study contributes to the establishment of a systematic causation model for analyzing the causes of coal mine accidents and helps form corresponding risk prevention measures directly and objectively.

## 1. Introduction

As the resource with the highest proportion in China’s energy consumption, the rapid growth of coal mine production guarantees the steady development of its national economy [1]. In recent years, the overall safety situation of China’s coal-mining industry has been progressively improving, with the mortality rate per million tons, the number of total deaths and the number of accidents showing a downward trend annually. In 2020, the mortality rate per million tons was only 0.058, which was the lowest in the safety records of China’s coal-mining industry [2]. However, serious accidents still occur from time to time, and there is still a huge gap compared with developed countries [3,4]. Of the 122 coal mine accidents that occurred in 2020 alone, ten were major accidents (death toll between 3 and 10) and three more were serious accidents (death toll between 10 and 30). Among them, on 27 September 2020, a fire accident occurred in Chongqing City, China, causing 16 deaths and 42 injuries, with a direct economic loss of 3.84 million dollars; on 4 December 2020, another fire accident occurred in Chongqing, resulting in 23 deaths and 1 serious injury, with a direct economic loss of 3.63 million dollars; and on 29 November 2020, a major flood accident occurred in Hunan Province, China, leading to 13 deaths and direct economic losses of 5.36 million dollars [2].

For the coal-mining industry, the specific causes of each accident are different. However, according to the analysis of accident investigation reports, the causes can be roughly divided into direct causes and indirect causes [5]. The direct causes refer to the unsafe behaviors or state of the operators who cause the accidents, which can be regarded as the first source of causes, while indirect causes are defects in internal management and external supervision, which can be taken as the second source. Thus, it can be inferred that coal mine accidents can mainly be attributed to human and organizational factors (HOFs). Under these circumstances, in order to better explain the mechanism of accidents from the perspective of human and management, accident-causing models, as important analysis tools, have been proposed and improved to adapt them to the development of sociotechnical systems.

The domino model was the first analytical model put forward to explicitly point out human error in industrial accidents [6]. The collapse of the first domino will knock down the subsequent dominoes one after another until injury occurs. Since the domino model oversimplifies the process of control over human behaviors in accidents, Bird, Adams and Weaver extended this model, and incorporated managerial decisions into accident causes [7,8,9,10]. Nevertheless, simple linear accident models of this kind have generally been considered too straightforward, with limited applicability in increasingly complex sociotechnical systems. Owing to the need for a more reasonable method and a stronger model for understanding accidents, simple linear models were gradually replaced by epidemiological models in the 1980s, which compared the events that lead to accidents with the spread of diseases, and held that accidents were the results of a variety of factors accidentally existing in both space and time. The most famous epidemiological model is the Swiss cheese model proposed by Reason, who considered that an accident involves four categories of factors (four pieces of cheese), namely, organizational influences, unsafe supervision, preconditions for unsafe acts, and direct unsafe acts [11]. Each piece of cheese represents a layer of the defense system, while the holes in the cheese represent loopholes or defects in the corresponding defense system. Since the positions and sizes of these holes are constantly changing, and when the holes in each piece of cheese are arranged in a straight line in a given instant, an accident-causing “trajectory” is formed, where the risks pass through the loopholes in all of the defense systems and eventually lead to accidents. However, this model does not present exact definitions of “holes” in each piece of cheese, thus limiting its practical application [12]. To facilitate the investigation and analysis of accidents, Shappell and Wiegmann, based on the Swiss cheese model, established the Human Factors Analysis and Classification System (HFACS) by analyzing hundreds of aviation accidents, clearly defining the “holes” in the cheese, and this has been widely used in various fields [13].

Since the development of this model, Baysari et al. identified the errors that frequently resulted in the occurrence of rail accidents under the guidance of HFACS framework, finding that slips of attention were the most common unsafe acts committed by drivers [14]. Rashid et al. performed a statistical analysis of 58 helicopter maintenance-induced safety occurrences, put forward the HFACS-ME model in maintenance extension, and studied the survivability and severity distribution of such occurrences [15]. Chauvin et al. applied the HFACS system to maritime collision accidents to analyze HOFs in the shipping industry, and found that most accidents were the result of decision errors [16]. Cohen et al., through the investigation of the causes in surgical near-miss events, discovered that most issues involved the preconditions for unsafe acts, followed by unsafe acts, organizational influences and unsafe supervision [17]. Chen put forward the HFACS-CI framework in the construction industry, and verified its effectiveness through concrete accident analysis [18]. Wang et al. revised the HFACS system for small- and medium-sized enterprises in the chemical industry, and considered that it could effectually identify and distinguish the causes in chemical accidents [19]. In the coal-mining industry, Patterson and Shappell analyzed 508 coal mine accidents in Australia, and the results indicated that skill-based errors in the HFACS model were the most common unsafe acts in miners [20]. Liu et al. collected over 300 coal mine accidents in China, established the HFACS-CM framework of China’s coal-mining industry, and calculated the weight of each causation factor [21].

However, previous scholars have mainly used research methods based on grounded theory, such as literature summary, on-the-spot investigation, in-depth interview, or manual analysis to identify unsafe factors in accidents. Under these circumstances, some important accident-causing factors or hidden variables are not recognized, and consequently, are not considered in the combinations of variables. Thus, the adaptability between assumptions and data as well as explanatory ability in traditional models will be weak. In addition, although HFACS framework, as a hierarchical model, has apparent causal relationships within layers, since there are many elements in the system, the correlation of unsafe elements between layers is often ignored, and the causality of specific elements that have an important role in restraining accidents is not recognized, that is, the transmission paths of the accident-causing “trajectory” are still unclear, which can limit the effectiveness of precautions in the coal mine risk management process. Based on this, by adopting the data-driven research paradigm, this study uses text mining technology to identify manifestations of accident-causing factors that restrict safe production in the coal-mining industry and maps them into the HFACS framework, forming a modified HFACS-CM in the coal-mining industry. Moreover, under the guidance of the inherent causal relationships in the system, frequent patterns of specific unsafe factors between layers are further discovered, forming a causal chain with clear unsafe elements in coal mine accidents, so as to upgrade the relevant accident precautions.

The remainder of this paper is organized as follows. Section 2 introduces the data sources, the basic concepts of each method, and the research framework. In Section 3, manifestations of the accident causes are extracted through text mining, and hierarchical association rules are mined using the Apriori algorithm under the guidance of the modified HFACS-CM framework. Finally, some concluding remarks are provided in Section 4.

## 2. Preliminaries and Research Framework

### 2.1. Data Set

**Figure 1:**
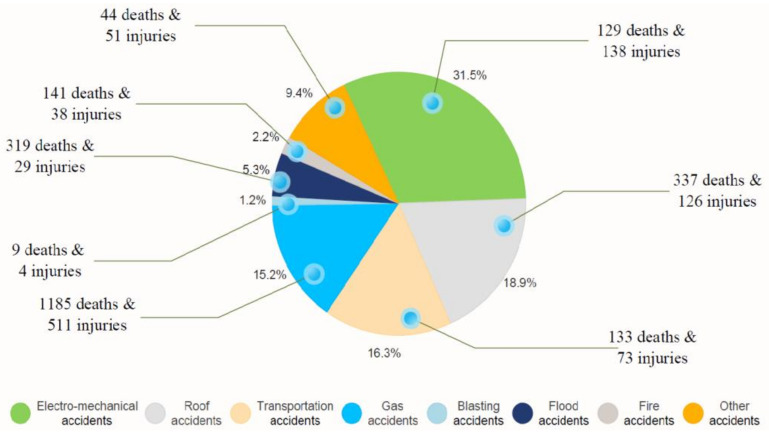
Statistics of 883 coal mine accidents.

Since accident investigation reports can reveal the causes of accidents in detail, they can also reveal mistakes within the process of risk management and the defects of various constructive documents in coal-mining enterprises. Therefore, accident investigation reports were used in this paper as the original data for text mining. In the selection of coal mine accident reports, some samples came from the State Administration of Work Safety, China’s Emergency Management Department, and the official website of the provincial and municipal safety supervision administration, while other samples were obtained through field investigation of China’s coal-mining enterprises. After data cleaning, a total of 883 accidents in the coal-mining industry in China from 2011 to 2020 were identified, including eight accident types: gas, electro-mechanical, transportation, flood, fire, blasting, roof, and other accidents. Specific information on the various types of accidents is shown in Figure 1.

### 2.2. HFACS Framework

**Figure 2:**
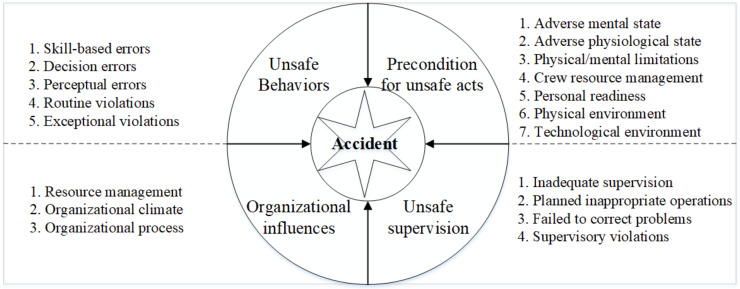
The original HFACS framework.

The original HFACS framework contained 4 categories and 17 subcategories. However, considering the influences of environment on unsafe behaviors, Wiegmann and Shappell added “physical environment” and “technical environment” into the category of “preconditions for unsafe acts” (Figure 2) [22]. After that, Patterson and Shappell, when analyzing coal mine accidents, introduced the fifth category “outside factors”, based on the original HFACS framework, and divided it into subcategories including “regulatory factors” and “other factors” [20]. Liu et al. refined “outside factors” into “management factors”, “policy factors”, “economic factors”, and “historical factors” [21].

Since the coal mine industry is a sociotechnical system with the interaction of technology, society and organizational influences, the increasing complexity of technology and the rapid development of society mean that the system is not only affected by internal fluctuations, but also by the external environment, including market competition, economic and political pressure. Therefore, on the basis of the original HFACS framework, this paper took “external influences” as the fifth category in this model.

### 2.3. Text Mining

**Figure 3:**
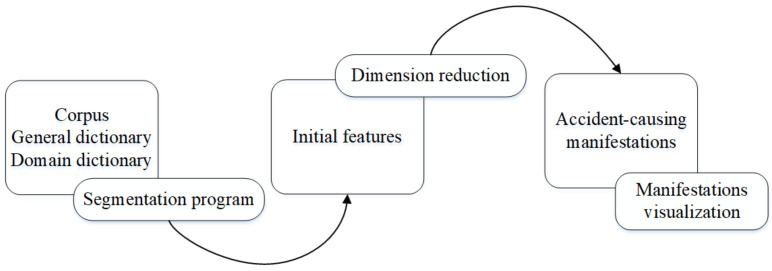
Process of manifestation extraction.

Text mining refers to the computer processing technology used to extract valuable information from text data, which can also be called knowledge discovery in a database [23]. In recent years, there has been a rising trend in the achievements related to text mining, and it is already becoming one of the most effective methods for studying the relationships between elements in various disciplines [24,25,26,27]. This paper mainly uses the function of text segmentation in text mining to extract the manifestations with the characteristics of 883 coal mine accidents. The process is illustrated in Figure 3.

### 2.4. Association Rules

Traditional models determine the combination of variables or constructs based on observation, theoretical deduction and empirical refinement, thus establishing theoretical assumptions, which are then tested through data demonstration. However, in the context of multidimensional data, numerous combinations of variables need to be examined, making difficult to build traditional models and test them one by one. At this time, it is necessary to use the association rules of data-driven paradigms to determine the correlation between variables in order to reduce the space and combination scale of the variables [28,29]. Nevertheless, correlations among large-scale data are not always accessible, since the traditional association algorithms are usually not perfect, that is, the rules generated usually contain some meaningless or even incorrect rules, referred to as weak rules and negative rules. Moreover, the general rules often fail to reflect the useful relationships among data, and thus cannot fully express deep knowledge in practical applications. In this context, this paper aims to discover the association patterns of unsafe factors under the guidance of the HFACS framework, which has its own causal and logical characteristics, giving the antecedents and consequents in the association rules a certain causality, such that the rules mined are strong and contain more valuable knowledge than traditional models.

At present, the commonly used association algorithms are the Apriori algorithm, the DIC algorithm, and the FP-Growth algorithm [30]. In this paper, to extract the frequent patterns of unsafe factors, the Apriori algorithm was used to find meaningful connections in multidimensional data sets and to improve inspection efficiency in coal-mining enterprises. In this algorithm, the correlation between elements is mainly reflected by three indicators: “support”, “confidence” and “lift” [31,32,33].

Let I = {$i_{1},i_{2},i_{3}\ldots ,i_{n}$} be defined as the set of items in all events, and let T = {$t_{1},t_{2},t_{3}\ldots ,t_{m}$} be the set of all events. Support count σ(X) = |{$X_{t_{i}},t_{i}\in T$}| represents the number of occurrences of a given item set in all transactions, and the support between two indicators can be expressed as follows:

(1) $$\mathrm{support}\left(X\;\&\;Y\right)=p\left(X\;\&\;Y\right)=\frac{\sigma \left(X\cup Y\right)}{N}$$

The confidence between indicators can be expressed as follows:

(2) $$\mathrm{confidence}\left(X\to Y\right)=\frac{\sigma \left(X\cup Y\right)}{\sigma \left(X\right)}$$

The value of lift between indicators can be expressed as follows:

(3) $$\mathrm{lift}\left(X\to Y\right)=\frac{\mathrm{confidence}\left(X\to Y\right)}{\mathrm{support}\left(Y\right)}$$

### 2.5. Research Framework

**Figure 4:**
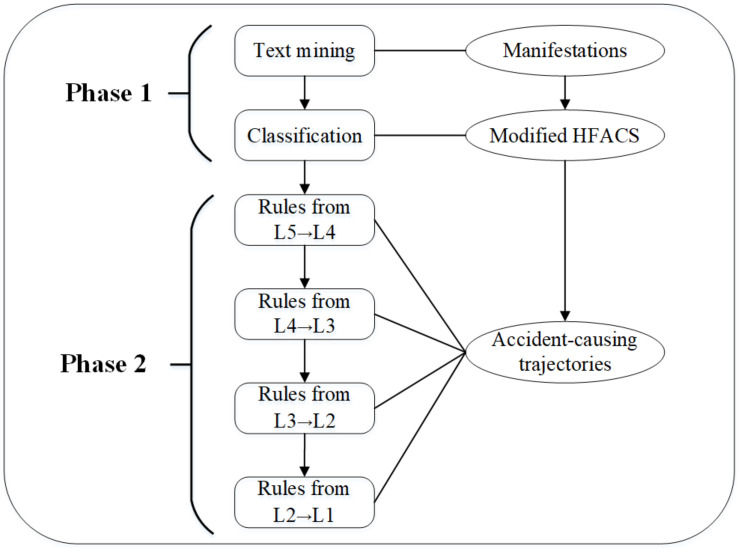
Research framework.

As shown in Figure 4, the research process of this paper includes two stages and four steps. The first stage contains the mining of manifestations of causes in coal mine accidents (Step 1) and the revision of HFACS framework in the coal-mining industry (Step 2). The second stage is the extraction of causal association rules among unsafe factors in different layers (Step 3) and the formulation of specific accident-causing trajectories (Step 4).

## 3. Results and Analysis

### 3.1. Text Mining Results

After initial text segmentation, there were as many as 3000 original items containing a lot of useless information that could seriously interfere with the subsequent analysis, so it was necessary to reduce the dimensions of the featured items. In this paper, the chi-squared statistic was used to extract useful features [34]. In addition, the calculation formula was:

(4) $$\chi^{2}\left(t,c_{i}\right)=\frac{n\times {\left(a\times b-c\times d\right)}^{2}}{\left(a+c\right)\left(b+d\right)\left(a+b\right)\left(c+d\right)}$$

In Equation (4), *n* represents the total amount of text; $c_{i}$ refers to the category of text; *a* is text frequency that belongs to $c_{i}$ and contains item *t*; *b* is text frequency that does not belong to $c_{i}$ but contains item *t*; *c* stands for the text frequency that belongs to $c_{i}$ but does not contain item *t*; *d* indicates the text frequency that does not belong to $c_{i}$ and does not contain item *t* either.

(5) $$\chi_{max}^{2}\left(t\right)=max_{i=1}^{m}\{\chi^{2}\left(t,\;c_{i}\right)\}$$

Most meaningless items can be removed by using the *m* value in Equation (5), so as to reduce the dimensions of characteristics. A total of 55 features representing different causes in coal mine accidents were finally obtained, as shown in Table 1, where F represents frequency.

### 3.2. The Modified HFACS-CM Framework

As the 55 manifestations cover different aspects of coal mine safety production, it is necessary to classify them with a suitable theoretical framework. As for human factor modeling and assessment, there are various techniques available, such as the Success Likelihood Index Method (SLIM), the Swiss Cheese Model, the Human Factors Analysis and Classification System (HFACS) and Standardized Plant Analysis Risk-Human Reliability Analysis (SPAR-H). Of all of these models, HFACS describes accident causes in more detail than the others and can help analyze multiple cases, enabling us to systematically excavate and categorize the direct causes of security failure with respect to coal mine accidents and the indirect causes behind them [11,12,35,36]. The process of mapping manifestations into HFACS was completed by the following three steps:

Step 1: Roughly categorize the manifestations. According to their nature, all manifestations were sequentially classified into the five categories of HFACS.

Step 2: Accurately map manifestations into HFACS in accordance with definitions of subcategories defined in the original HFACS framework. During this process, the identified manifestations were respectively mapped into the subcategories in the model. Since there are no manifestations in the 55 featured above that fit “physical/mental limitations”, this subcategory was removed. In addition, the descriptions of employees’ behavioral violations were vague in selected accident reports. For example, in one of the roof accidents, when analyzing the direct causes, it was disclosed in the accident report that “Some worker stood on coal gangue, operating against rules, caused the coal gangue to fall and was buried eventually”, making it hard to distinguish whether it was an “exceptional violation” or a “routine violation”, so the two types of violations were unified as “violations”. Additionally, since there is no category of “external influences” in the original HFACS model, subcategories were put forward according to the connotations of all of the manifestations above and the classification of previous studies, so the category of “external influences” was further divided into “policy factors” and “management factors” [20,21]. Consequently, 55 manifestations were split into 19 subcategories.

Step 3: Merge comparable manifestations. Under the same subcategory, if there are multiple causal factors expressing a similar concept, they can be grouped together. For example, factors such as “defects in roof management”, “defects in transportation management”, “defects in vehicle management”, “defects in equipment management” and “defects in ventilation management” are all caused by defective regulations applicable to a specific management department, so they were merged as “unsound sectional regulations”. Likewise, “inadequate performance assessment” and “imperfect aggregate regulations” refer to defective norms applicable to all employees and departments in coal mines, and were consolidated as “unsound integral regulations”. In addition, if there are inclusion relationships among manifestations, they should be merged as one. Therefore, “equipment against regulations” was merged into “unqualified machinery”; “lack of guard lines” together with “unreasonable working face layout” were merged to “disorganized workplace”; “lack of safety confirmation” was incorporated into “operation at risk”; “lack of self and mutual protection awareness” was merged into “weak safety awareness of employees”; “lack of specified equipment” was incorporated into “lack of equipment”; “insufficient staffing” and “inadequate safety supervision” were incorporated into “unreasonable labor organization” and “failure to intervene in unsafe acts”, respectively. Finally, some manifestations were re-expressed to reveal the essential causes of coal mine accidents more clearly. “Lack of preshift meetings” was modified to “unseasonable work arrangement” and “lack of communication in shift change” was changed to “miscommunication”.

**Figure 5:**
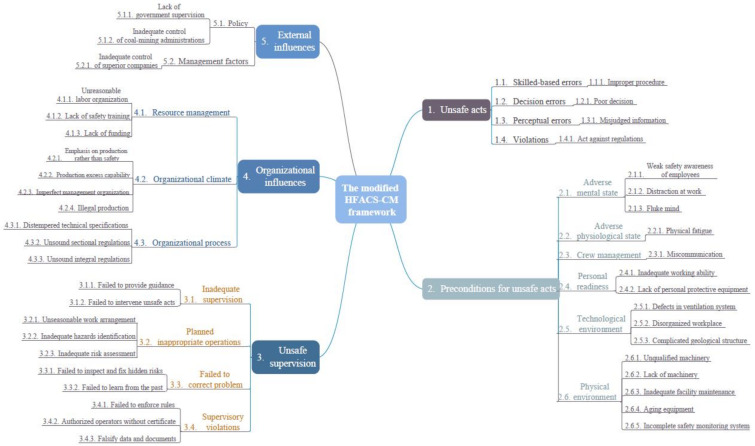
The modified HFACS-CM framework.

After identifying and mapping all manifestations, the HFACS-CM framework was formed, containing 42 accident-causing factors. In this framework, organizational influences contained 10 factors. Unsafe supervision contained 10 factors. Preconditions for unsafe acts contained 15 factors. Unsafe acts of operators contained 4 manifestations, and external influences contained 3 factors. The modified HFACS-CM framework is illustrated in Figure 5.

### 3.3. Causal Association Rules

Text segmentation can extract common factors that cause various accidents, but cannot discover the intrinsic relations. However, accidents are the result of multiple unsafe factors spreading in the accident chain, so determining the progressive modes of unsafe factors in HFACS-CM framework can systematically reveal patterns of factors involved in past coal mine accidents. To further clarify interactions among different contributing factors, this paper used the Apriori algorithm to mine the rules and trace the accident-causing paths from external influences to employees’ unsafe acts layer by layer. When preprocessing data, 0–1 coding was used to convert the causative factors in text form into digital representation. If an accident corresponds to a specific factor, it is recorded as 1, otherwise as 0. Therefore, the accident database was converted into a matrix consisting of 0’s and 1’s.

#### 3.3.1. Causal Rules from External Influences to Organizational Influences (L5→L4)

**Figure 6:**
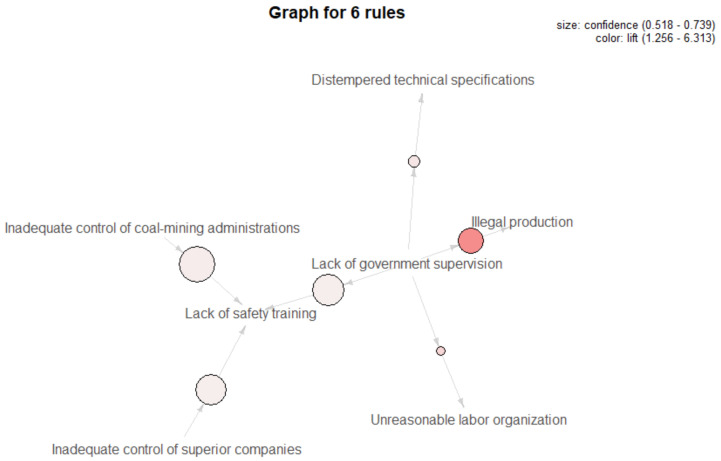
Visualization of causal rules between L5 and L4.

Based on a minimum support degree of 0.01, a minimum confidence degree of 0.5 and a minimum lift degree of 1.0, the antecedents of the rules were set as factors in external influences, and the consequents were factors in organizational influences. Relationships of 13 unsafe elements in two layers were carried out, and the causal rules are shown in Table 2. The visualization of six strong association rules is presented in Figure 6, where bubbles indicate the existence of association rules, the size of bubbles represents the value of confidence, and the gradation of color stands for lift value, revealing the degree of connection between factors.

Specifically, it is apparent that most of the rules are linked to government supervision (Rules 3–6). If the government fails to supervise safe production, it can lead to a poor quality of safety education and training, illegal production, technical specifications that do not meet the requirements, and unreasonable labor organization in coal-mining enterprises, among which government supervision and enterprises’ illegal production have the highest lift degree (6.31), indicating the strongest correlation between them. In addition, apart from government supervision, the poor control of coal-mining administrations and parent companies allows enterprises to conduct inadequate safety training since 12% and 11% cases in all accidents were caused by them respectively.

#### 3.3.2. Causal Rules from Organizational Influences to Unsafe Supervision (L4→L3)

**Figure 7:**
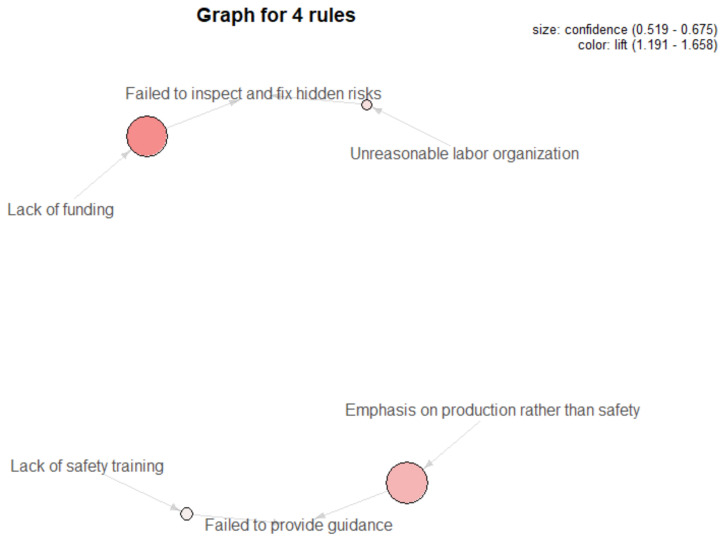
Visualization of causal rules between L4 and L3.

Under the same working environment, the association patterns of 23 unsafe factors under the layers of unsafe supervision and organizational influences were mined, with the results shown in Table 3 and Figure 7. It can be seen that the co-occurrence frequency of inadequate safety training and failure to provide guidance in Rule 1 is as high as 29%, and by the confidence value (0.53), it can be concluded that when the safety training for employees is not in place, there is over a 50% chance that supervisors will deliver commands against rules, while when the organizational climate in companies emphasizes production over safety, the possibility of illegal commands increases to 68%, and the lift degree changes from 1.19 to 1.51. Moreover, Rule 2 and Rule 4 indicate that supervisors failing to inspect and fix hidden risks are likely to be triggered by unreasonable labor organization or lack of funding, which has a stronger influence on the quality of hidden risks investigation in coal-mining enterprises.

#### 3.3.3. Causal Rules from Unsafe Supervision to Unsafe Preconditions (L3→L2)

**Figure 8:**
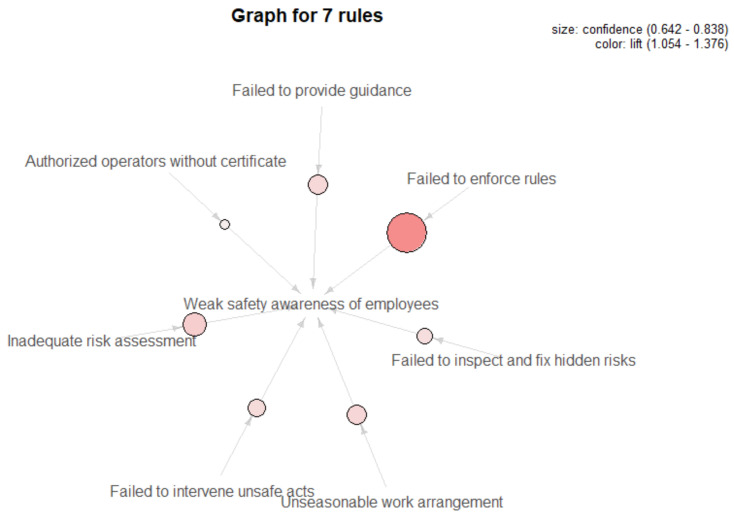
Visualization of causal rules between L3 and L2.

Continuing to progressively dig down into the rules between unsafe supervision and unsafe preconditions, as shown in Table 4 and Figure 8, all causal rules concern employees’ ideological states. Among them, failing to provide guidance or intervene in unsafe acts, inadequate investigation and rectification of hidden risks, unreasonable work arrangement at preshift meetings, failure of leaders to strictly implement relevant regulations, permission of supervisors for unqualified crew to operate, and inadequate hazard identification will all lead to unsafe mental states among employees such as insufficient awareness of self-protection and mutual protection or a weak sense of responsibility. Combined with the visual map of the rules, it can be seen that although the co-occurrence of supervisors’ illegal commands and employees’ weak safety awareness is the highest, when leaders fail to strictly implement relevant regulations, there is a more than 80% possibility that the safety awareness of front-line operators will be weak, and the correlation between them is also the strongest.

#### 3.3.4. Causal Rules from Unsafe Preconditions to Unsafe Acts (L2→L1)

**Figure 9:**
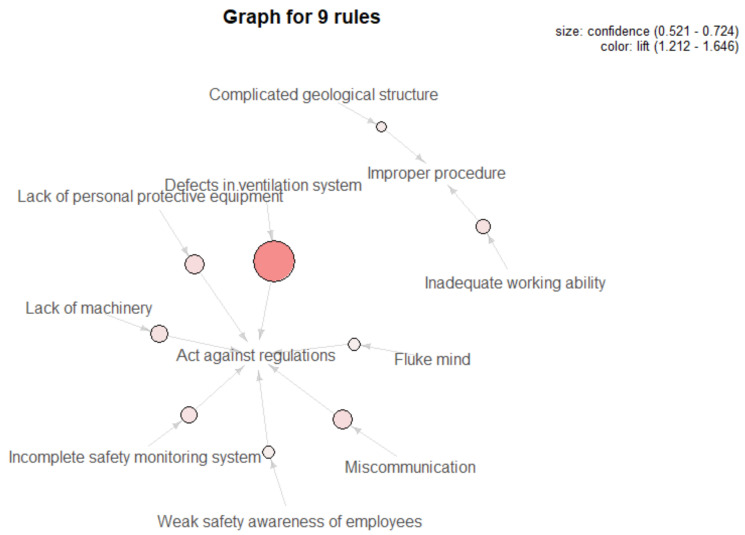
Visualization of causal rules between L2 and L1.

Finally, the association algorithm was used to mine useful knowledge under unsafe preconditions and direct unsafe acts, and the results are presented in Table 5 and Figure 9. Through analysis, it is found that the majority of unsafe factors in unsafe preconditions have a direct influence on operators’ unsafe acts. In particular, front-line operators’ weak safety awareness, fluke mind, not wearing personal protective equipment or poor communication during shift change can all result in violations during operation. In addition, unsafe state of technological and physical environment can also bring about employees acting against rules, such as lack of equipment and defective safety monitoring systems and ventilation systems, and when the design of ventilation systems in coal-mining enterprises is unreasonable, the possibility of employees acting illegally goes up to 72%, with the highest correlation, while skill-based errors like improper procedures are mainly caused by workers’ inadequate working ability or experience and complex geological structures.

#### 3.3.5. Accident-Causing Trajectories of Key Factors in the HFACS-CM Framework

**Figure 10:**
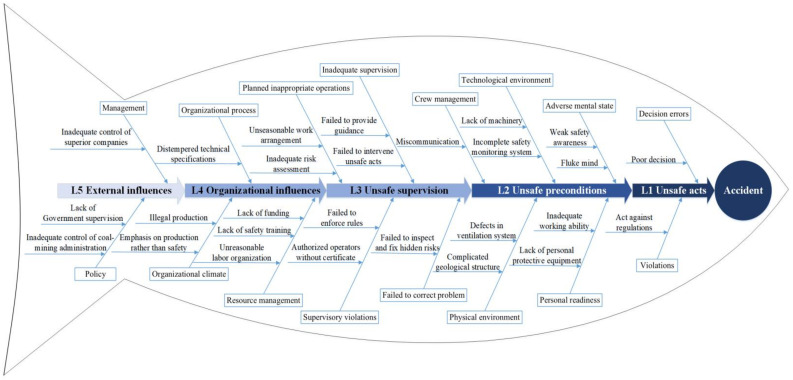
The adapted fishbone diagram about the key contributing factors in coal mines.

**Figure 11:**
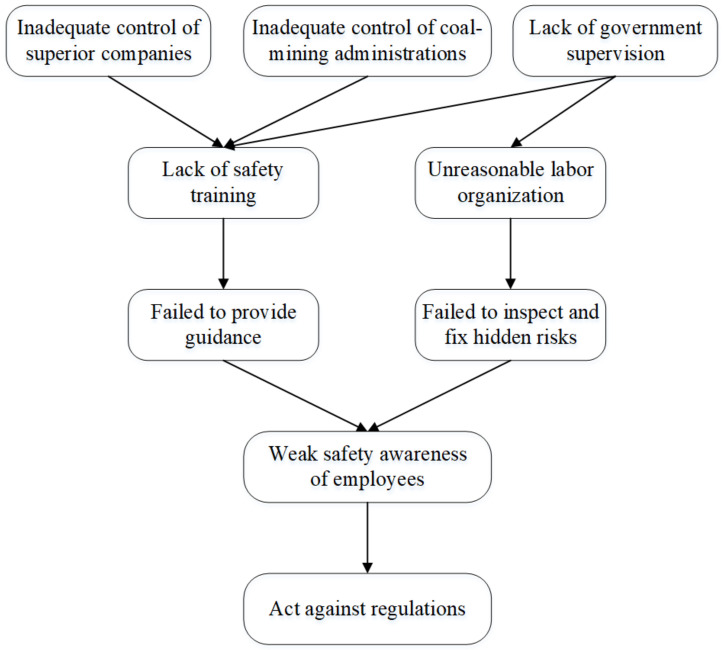
Accident-causing trajectories of specific factors in HFACS-CM framework.

According to the unsafe factors in the causal association rules above, the adapted fishbone diagram was used to list the key accident-causing factors in HFACS-CM framework, as shown in Figure 10, where the “eye” of the fish represents the occurrence of accidents, and the main bone, which is located at the diagram axis, consists of five arrows with graduated colors in accordance with the five layers in the HFACS-CM framework. For the fishbone diagram, key causation factors of coal mine accidents were added to fill in the bones of the fish. Accordingly, the accident-causing trajectories composed of specific factors were formed (Figure 11). It can be clearly seen that there are four main paths for the formation of coal mine accidents. In the first place, ineffective supervision by superior companies, coal mine administrations and the government can initially affect the quality of safety education and training in coal-mining enterprises, which will further lead to illegal commands by supervisors, where the safety awareness of front-line operators will gradually fade, and eventually illegal operations being conducted, directly causing accidents. Beyond that, the government’s ineffective supervision can also lead to a company’s unreasonable labor organization, such as insufficient staffing and unclear distribution of responsibilities, which can directly influence the quality of investigation and rectification towards hidden risks, gradually bringing about a long-term decline in employees’ vigilance against hazards or ability to assess risks, and conducting acts against rules.

In view of the accident routes in the process of coal mine safety management, this paper puts forward the following suggestions.

(1) First, the government, coal-mining administrations, and superior companies need to strengthen the guidance and supervision with respect to the quality of enterprises’ safety training and education, as well as using science-based approaches to ensure that accidents are effectively managed and positive learning is achieved [37]. Apart from that, it is also crucial for government departments to formulate censorship regulations so as to regularly check whether the enterprise’s labor organization is appropriate.
(2) Second, when organizing safety education and training, coal-mining enterprises should increase attention devoted to the examination of mastery of safety knowledge among supervisors, improving their work efficiency, putting an end to illegal commands, and ensuring that they can make the correct decisions in coal mine production. In addition, a department for investigating and rectifying hidden risks should be set up with adequate staff, so as to ensure regular inspection and timely correction of hidden risks can be implemented throughout the whole process of production.
(3) Finally, for ordinary workers, a safety education and assessment system should be established to help them conduct regular examinations of their awareness of self-protection and mutual protection, legal production awareness, and their ability to identify hazards, so as to expose the weaknesses of employees’ safety awareness and develop targeted solutions for different situations.

## 4. Conclusions and Discussion

The aim of this study was to reveal the accident-causing trajectories in China’s coal mine industry by using data-driven methods instead of traditional grounded-theory-based approaches. Theoretically, grounded theory starts with the actual observation, emphasizing the generalization of experience from the original data to a systematic theory. However, this qualitative method has its shortcomings. Firstly, since the data collection is based on the personal observation of researchers, the objectivity of the conclusions is limited. Secondly, when the data are massive and complicated, the burden for “human brains” to analyze is heavy and the accuracy and reliability will accordingly be reduced. Thus, this paper adopted a data-driven approach, which, through the statistical analysis of the whole body of data with the help of different algorithms, aimed to find correlations among the data objectively and to use them to guide decision making directly. Overall, through data-driven methods, unsafe human factors and management factors that restrict safe production in coal mines were found, and the causal association rules among unsafe factors were extracted layer by layer to reveal the causal paths of specific factors that can lead to coal mine accidents. The following conclusions are drawn.

(1) With text segmentation technology to extract feature words with accident-causing characteristics of the coal mine accidents, a total of 55 manifestations were obtained. Then, according to the nature of each manifestation, they were mapped into the HFACS framework, forming a revised HFACS-CM model composed of 5 categories, 19 subcategories and 42 specific factors for coal-mining industry.
(2) In the process of mining progressive modes among external influences, organizational influences, unsafe supervision, unsafe preconditions and unsafe acts, it was found that the ineffective supervision of the government and other external supervisory departments could bring about the unsafe state of internal management such as insufficient safety training and unreasonable labor organizations, which will continually cause supervisors to issue illegal commands and to fail to investigate or rectify hidden risks, thus leading to weak safety awareness among employees and behavioral violations.

This study contributes to establishing a systematic causation model for analyzing the causes of the failure of security to prevent coal mine accidents in China. It introduces the HFACS-CM framework for systematically and quantitatively analyzing the underlying human, organizational, environmental and technical factors. Such a model can facilitate more comprehensive accident investigation and form targeted precautions directly. This study also enriches existing risk analysis methods in the field of safety management by extending the application of HFACS and text mining along with association algorithm to expose effective causation rules among unsafe factors instead of general and weak rules.

The limitations of the current study should also be acknowledged, and these suggest directions for further research. The limitations mainly lie in the setting of the HFACS model. Firstly, with the development of accident analysis tools, there exist some nonlinear accident causation models that are more advanced for the tightly coupled and nontractable socio-technical systems compared with complex-linear causation model such as HFACS [38]. In addition, for unsafe factors that do qualify for the accident-causing trajectories presented, when accidents are seen as complex phenomena, there may not be an obvious relationship between the behavior of parts in the system and system-level outcomes, which is the weakness of the HFACS model [39]. Under these circumstances, complexity theory should be taken into account for further study. Secondly, if there are real links between accident-causing factors that do in reality have a negative impact, but their correlation does not align with the HFACS framework, then some real meaningful rules will be left out. Thirdly, due to the nature of this study, there will be great variability between accident descriptions and bias in data processing [40]. The method of grouping factors together when modifying the HFACS-CM framework will inevitably influence the taxonomy, which is the central mechanism in HFACS. For example, if factors are merged with a less detailed level of granularity, the association rules generated may not contain too much useful information. Overall, these limitations suggest the need to think beyond the HFACS model itself, considering the complexity of accident occurrence mechanism as well as the combination of data-driven results with actual facts.

## Figures and Tables

**Table 1:** Fifty-five manifestations extracted from coal mine accidents.

Item	Manifestations	F	Item	Manifestations	F
1	Inadequate control of superior companies	492	29	Incomplete safety monitoring system	37
2	Failure to provide guidance	423	30	Defects in ventilation system	35
3	Weak safety awareness of employees	366	31	Inadequate safety supervision	28
4	Distempered technical specifications	329	32	Distraction at work	27
5	Failure to inspect and fix hidden risks	289	33	Unreasonable labor organization	26
6	Act against regulations	193	34	Inadequate risk assessment	24
7	Lack of safety training	173	35	Failed to learn from the past	21
8	Inadequate hazards identification	168	36	Lack of personal protective equipment	20
9	Failure to enforce rules	156	37	Falsified data and documents	16
10	Lack of government supervision	152	38	Fluke mind	14
11	Inadequate coal-mining administration control	140	39	Unreasonable working face layout	12
12	Illegal production	128	40	Defects in transportation management	12
13	Improper procedure	87	41	Production excess capability	12
14	Insufficient staffing	74	42	Lack of funding	7
15	Lack of preshift meetings	73	43	Lack of guard lines	5
16	Lack of safety confirmation	70	44	Emphasis on production rather than safety	4
17	Inadequate working ability	69	45	Aging equipment	4
18	Operation at risk	65	46	Defects in equipment management	3
19	Imperfect aggregate regulations	64	47	Defects in ventilation management	3
20	Lack of self and mutual protection awareness	59	48	Physical fatigue	3
21	Complicated geological structure	56	49	Lack of equipment	2
22	Imperfect management organization	52	50	Disorganized workplace	1
23	Authorized operators without certificate	49	51	Unqualified machinery	1
24	Defects in roof management	46	52	Equipment against regulations	1
25	Misjudgment of information	40	53	Defects in vehicle management	1
26	Inadequate facility maintenance	40	54	Lack of specified equipment	1
27	Failure to intervene in unsafe acts	38	55	Inadequate performance assessment	1
28	Lack of communication in shift change	38			

**Table 2:** Causal rules between external influences and organizational influences.

Number	Rules	Support	Confidence	Lift
1	{Inadequate control of coal-mining administrations} => {Lack of safety training}	0.12	0.74	1.34
2	{Inadequate control of superior companies} => {Lack of safety training}	0.11	0.69	1.26
3	{Lack of government supervision} => {Lack of safety training}	0.07	0.70	1.26
4	{Lack of government supervision} => {Illegal production}	0.06	0.65	6.31
5	{Lack of government supervision} => {Distempered technical specifications}	0.05	0.54	1.88
6	{Lack of government supervision} => {Unreasonable labor organization}	0.05	0.52	2.93

**Table 3:** Causal rules between organizational influences and unsafe supervision.

Number	Rules	Support	Confidence	Lift
1	{Lack of safety training} => {Failed to provide guidance}	0.29	0.53	1.19
2	{Unreasonable labor organization} => {Failed to inspect and fix hidden risks}	0.09	0.52	1.29
3	{Emphasis on production rather than safety} => {Failed to provide guidance}	0.03	0.68	1.51
4	{Lack of funding} => {Failed to inspect and fix hidden risks}	0.01	0.67	1.66

**Table 4:** Causal rules between unsafe supervision and unsafe preconditions.

Number	Rules	Support	Confidence	Lift
1	{Failed to provide guidance} => {Weak safety awareness of employees}	0.31	0.70	1.16
2	{Failed to inspect and fix hidden risks} => {Weak safety awareness of employees}	0.28	0.68	1.12
3	{Failed to intervene unsafe acts} => {Weak safety awareness of employees}	0.18	0.70	1.14
4	{Unseasonable work arrangement} => {Weak safety awareness of employees}	0.17	0.71	1.16
5	{Failed to enforce rules} => {Weak safety awareness of employees}	0.10	0.84	1.38
6	{Authorized operators without certificate} => {Weak safety awareness of employees}	0.07	0.64	1.05
7	{Inadequate risk assessment} => {Weak safety awareness of employees}	0.03	0.73	1.20

**Table 5:** Causal rules between unsafe preconditions and unsafe acts.

Number	Rules	Support	Confidence	Lift
1	{Weak safety awareness of employees} => {Act against regulations}	0.32	0.53	1.21
2	{Inadequate working ability} => {Improper procedure}	0.11	0.55	1.30
3	{Lack of personal protective equipment} => {Act against regulations}	0.08	0.58	1.32
4	{Complicated geological structure} => {Improper procedure}	0.07	0.52	1.23
5	{Lack of machinery} => {Act against regulations}	0.06	0.57	1.29
6	{Miscommunication} => {Act against regulations}	0.05	0.58	1.32
7	{Incomplete safety monitoring system} => {Act against regulations}	0.04	0.56	1.28
8	{Defects in ventilation system} => {Act against regulations}	0.02	0.72	1.65
9	{Fluke mind} => {Act against regulations}	0.02	0.53	1.21

## Data Availability

The data presented in this study are available on request from the corresponding author. The data are not publicly available due to privacy restrictions.
